# Late-Life Blood Pressure and Cerebral Amyloid Angiopathy: Findings from the U.S. National Alzheimer’s Coordinating Center Uniform Dataset

**DOI:** 10.3390/neurolint16040061

**Published:** 2024-07-29

**Authors:** Mo-Kyung Sin, N. Maritza Dowling, Jeffrey M. Roseman, Ali Ahmed, Edward Zamrini

**Affiliations:** 1 College of Nursing, Seattle University, Seattle, WA 98122, USA; 2 Department of Acute & Chronic Care, School of Nursing, George Washington University, Washington, DC 20147, USA; nmdowling@gwu.edu; 3 Department of Epidemiology & Biostatistics, Milken School of Public Health, George Washington University, Washington, DC 20147, USA; 4 Department of Epidemiology, University of Alabama at Birmingham, Birmingham, AL 35294, USA; bush@uab.edu; 5 Center for Data Science and Outcomes Research, Veterans Affairs Medical Center, Washington, DC 20242, USA; ali.ahmed@va.gov (A.A.); ezamrini@irvineclinical.com (E.Z.); 6 Department of Medicine, School of Medicine & Health Sciences, George Washington University, Washington, DC 20052, USA; 7 Department of Medicine, School of Medicine, Georgetown University, Washington, DC 20057, USA; 8 Biomedical Informatics Center, School of Medicine & Health Sciences, George Washington University, Washington, DC 20052, USA

**Keywords:** late-life blood pressure, Braak, CERAD, cerebral amyloid angiopathy, *APOE* e4

## Abstract

High blood pressure (BP) and cerebral amyloid angiopathy (CAA) are two common risk factors for intracranial hemorrhage, potentially leading to cognitive impairment. Less is known about the relationship between BP and CAA, the examination of which was the objective of this study. We analyzed data from 2510 participants in the National Alzheimer’s Coordinating Center (NACC) who had information on longitudinal BP measurements before death and on CAA from autopsy. Using the average of four systolic BPs (SBPs) prior to death, SBP was categorized into three groups: <120 mmHg (*n* = 435), 120–139 mmHg (*n* = 1335), and ≥140 mmHg (*n* = 740). CAA was diagnosed using immunohistochemistry in 1580 participants and categorized as mild (*n* = 759), moderate (*n* = 529), or severe (*n* = 292). When adjusted for age at death, sex, *APOE* genotype, Braak, CERAD, antihypertensive medication use, and microinfarcts, the odds ratios (95% CIs) for CAA associated with SBPs of 120–139 and ≥140 mmHg were 0.91 (0.74–1.12) and 1.00 (0.80–1.26), respectively. Findings from predictor effect plots show no variation in the probability of CAA between the three SBP categories. Microbleeds had no association with CAA, but among those with SBP ≥ 130 mmHg, the proportion of those with microbleeds was numerically greater in those with more severe CAA (*p* for trend, 0.084). In conclusion, we found no evidence of an association between SBP and CAA. Future studies need to develop non-invasive laboratory tests to diagnose CAA and prospectively examine this association and its implication on the pathophysiology and outcome of Alzheimer’s disease.

## 1. Introduction

Cerebral amyloid angiopathy (CAA) is present in about 80–90% of people with Alzheimer’s disease (AD) [1,2,3] and about 30% of older adults without AD or other neuropathological abnormalities [4]. Although affecting different regions (CAA is mostly in the cortex vs. hypertension is mostly in the subcortex), CAA and late-life hypertension are the most common risk factors of spontaneous intracranial hemorrhage in older adults, contributing to cognitive impairment. For example, a study reported that 51% (54/105) of people with CAA developed an intracranial hemorrhage, which was related to CAA severity: 87% (47/54) of intracranial hemorrhages happened in CAA severity grades 2–4 vs. 13% (7/47) in grade 1 CAA severity [5]. CAA, defined as amyloid β build-up in the walls of the cerebral blood vessels, can gradually weaken the vessels and make microscopic cracks or fissures that cause life-threatening lobar hemorrhages [6]. An intracranial hemorrhage can lead to cognitive impairment [7,8]. Possible causes include direct brain tissue damage, adjacent structural damage, ischemia from mass effect and the compression of brain tissue, and neurodegeneration associated with brain injury-related inflammation [9,10]. Hypertension is the most common cause of intracranial hemorrhages. Like CAA, chronic hypertension also causes small arterial-wall damage, leading to intracranial hemorrhage [11]. For example, the Perindopril Protection Against Recurrent Stroke Study reported that lowering the systolic blood pressure (SBP) resulted in a reduction in intracranial hemorrhages by 70% [11]. Hypertension promotes early endothelial dysfunction and blood–brain barrier breakdown, resulting in hypertensive arteriopathy-related small vessel-wall damage, leading to an accelerated formation of CAA in rats [12]. Increased pulse pressure (PP), from vascular stiffness and loss of vessel elasticity, results from structural changes in the arteries and reflects the chronicity of hypertension [11]. Increased PP has a demonstrated association with an elevated cortical amyloid burden [13,14], but limited studies have examined PP as a risk factor for cognitive impairment and dementia.

Because both CAA and hypertension are common in aging, associated with neurologic impairment, and potentially preventable, it is critical to understand any interactions between the two to understand cognitive impairment and dementia in older adults. However, we have limited empirical studies examining the relationship between late-life BP and CAA in human beings. The purpose of this autopsy-based study is to examine this relationship in a national cohort of older adults.

## 2. Methods

### 2.1. Data and Participants

**Figure 1:**
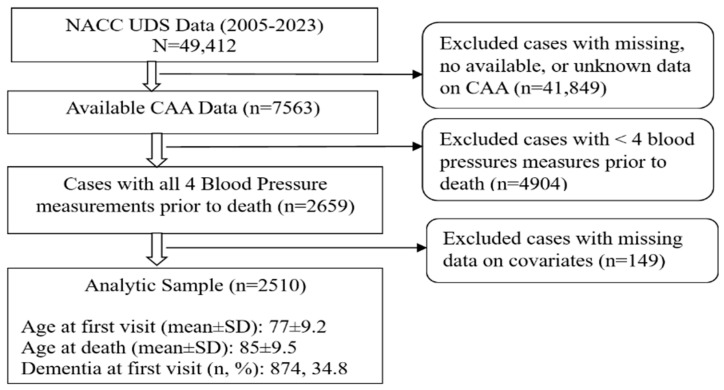
Flowchart of participant inclusion.

We analyzed data from the National Alzheimer’s Coordinating Center (NACC) that was collected by National Institute on Aging (NIA)-funded Alzheimer’s Disease Research Centers (ADRCs). Established in 1999 by the NIA/NIH (U01 AG016976) to facilitate collaborative AD research, the NACC functions as the centralized data repository for standardized clinical, genetic, and neuropathological data collected by the approximately 37 former and active ADRCs [15]. ADRCs aim to translate research advances into improved diagnosis and care for people with Alzheimer’s disease and related dementias and search for ways to treat and prevent these diseases. ADRCs enroll participants who are healthy, at risk, and have dementia, and they are recruited as a referral-based or volunteer case series. NACC recruitment and data collection have been previously described [16]. The Uniform Data Set (UDS) includes multi-domain standardized neurocognitive and phenotypic data, collected annually since 2005. The Neuropathology Data Set contains autopsy data for a subset of the UDS that is collected using a standardized neuropathological evaluation. Consent to autopsy is voluntary, but some ADRCs require autopsy consent before being accepted for the UDS. For our study, we started with the 2005–2023 UDS data (N = 49,412) and then excluded cases with missing, not available, or unknown data on CAA variables, resulting in N = 7563 with the available CAA data. We further excluded cases with less than 4 BP measurements prior to death and missing data on covariates, leading to an analytic sample of N = 2510 (see Figure 1). The time gap between the last BP and death was 3.029 years with a standard deviation of 2.318 for the 2510 analytic sample. Written informed consent was obtained from all participants or co-participants/caregivers of participants when the participants were cognitively impaired. Inclusion criteria for this study were the observed CAA data, with all available 4 BP measurements prior to death, and all the observed data on covariates such as the age at death, sex, antihypertensives, *APOE* e4, CERAD, Braak, and microinfarcts. Excluded cases are listed in Figure 1.

### 2.2. Identification of Cases and Non-Cases

Neuropathologic data were obtained from NACC’s Standardized Neuropathology Form [17,18]. The presence of autopsy-based CAA was determined using an immunohistochemical method [19], and its severity was ranked on a four-point scale: none, mild, moderate, or severe. Cases were defined by the presence of CAA in the brain. Those without any CAA were considered non-cases.

### 2.3. Study Exposure

BP was measured at annual visits following the NACC standard protocol [18]. In this study, we used the last four observed BPs measured prior to death. We looked at the SBP and PP (PP = SBP − DBP). We followed the Joint National Committee on Prevention, Detection, Evaluation, and Treatment of High Blood Pressure [20] guidelines for high BP to maintain consistency of the longitudinal nature of BP. The SBP was categorized into three levels for analyses: ≥140 mmHg, 120–139 mmHg, and <120 mmHg. PP was categorized into two groups: high (≥50 mmHg) and normal (<50 mmHg).

### 2.4. Covariates

The covariates included the age at death, sex, antihypertensive use, *APOE* e4, CERAD and Braak stages, and microinfarcts. The clinician or ADRD staff obtained detailed demographic, medical history, and medication use records at the baseline and annual visits using a standardized questionnaire. Data on the use of antihypertensive medications were binary (1 = yes; 0 = no) and were collected by the clinician or ADRC staff to determine whether or not the participant was taking any prescribed BP medication within the two weeks before the current visit. We estimated the proportion of hypertension medication use across all visits prior to death. The *APOE* e4 status was coded as “0 = absence of e4 allele” or “1 = 1 or 2 copies of e4 allele”.

The CERAD and Braak stages and microinfarcts were obtained from neuropathology data. The CERAD staging score was measured based on the semiquantitative estimates of neuritic plaque density under a microscope and categorized as “0 = none”, “1 = sparse”, “2 = moderate”, or “3 = frequent” [21]. The Braak stage, to classify the spread of neurofibrillary tangles, was measured in postmortem brain tissue under a microscope and categorized as “0 = stage 0 (normal)”, “1 = stage I (very mild)”, “2 = stage II (mild)”, “3 = stage III (moderate)”, “4 = stage IV (moderately severe)”, “5 = stage V (severe)”, or “6 = stage VI (very severe)” [22]. The presence of microinfarcts, defined as “a focal lesion attributed to ischemia, found only on microscopic examination, and judged by neuropathologist to be temporally remote” [23] was categorized as yes and no.

### 2.5. Statistical Analyses

Participant characteristics were summarized using means and standard deviations for continuous variables and counts and percentages for categorical variables. We compared patient characteristics at death among four CAA groups: none, mild, moderate, and severe. We conducted Pearson’s chi-square tests to evaluate the association between each categorical characteristic and CAA severity. The association between continuous predictors (e.g., proportion of antihypertensive medication use) and CAA was evaluated with F-tests.

Multivariable proportional odds logistic regression for ordered category outcomes [24,25] was used to examine the associations of CAA stages and BP measures. The proportional odds assumption for the model was assessed with the Brant–Wald test [26]. The Lipsitz likelihood-ratio (LR) [27] goodness-of-fit test was used to assess the overall adequacy of the model.

Both SBP and PP were modeled as the main exposures. We controlled for sex, age at death, *APOE* genotype, Braak stage, CERAD stage, antihypertensive medications, and microinfarcts. All analyses were carried out with R version 4.3.2 [28] and the polr and effects functions in the MASS package [29]. Statistical significance was determined at *p* < 0.05. In a secondary analysis, we also descriptively explored the association between microbleeds and SBP by CAA severity using cross-tabulation (contingency table) analysis. Then, a Jonckheere–Terpstra test [30,31] was used to observe any statistical significance.

## 3. Results

### 3.1. Participant Characteristics

Table 1 shows the characteristics of the study participants in total and stratified by CAA pathology: none, mild, moderate, or severe. The analytical sample had a total of 2510 participants with a known status of CAA. This cohort had at least four measures of their BP prior to their death. The average time gap between the last BP to death was 3.03 years (SD = 2.32). CAA was present in 1580 (62.9%) participants; 759 (30.2%) mild, 529 (21.1%) moderate, and 292 (11.6%) severe. Severe CAA was highly prevalent among *APOE* e4 carriers compared to non-carriers (62.7% vs. 37.3%), severe Braak stages compared to milder stages (V–VI: 77.7% vs. 0–II: 6.5%), and CERAD frequent neuritic plaque pathology scores compared to absence of plaques (frequent: 70.9% vs. absent: 4.1%) (see Table 1). There were statistically significant differences in the sex (*p* = 0.012), age at death (<0.001), *APOE* e4 (*p* < 0.001), Braak stage (*p* < 0.001), CERAD (*p* < 0.001), and proportion of antihypertensive medication use (*p* < 0.001) between individuals across all CAA pathology stages.

### 3.2. Blood Pressure and CAA

The results of the multivariable proportional odds logistic regression are shown in Table 2. The Brant–Wald test supported the proportionality assumption of the proportional odds model for ordinal logistic regression. The Lipsitz goodness-of-fit test also produced a good model fit (likelihood ratio: LR = 14.49; *p* = 0.11).

Compared to late-life mean SBP < 120 mmHg, SBP ≥ 140 mmHg was not associated with CAA stage severity (aOR = 1.08; 95% CI: 0.816–1.44). Our secondary analysis indicated that the risk of microbleeds increased with the severity of CAA (5.46%, 7.67%, and 10.73% in mild, moderate, and severe CAA, respectively) when hypertension (SBP ≥ 130 mmHg) was present (see Supplemental Table S1). However, there was no statistically significant trend of higher CAA severity with the presence/absence of microbleeds in the group with higher late-life mean SBP (≥130 mmHg) (TJT = 79,379; z = 1.725; *p* = 0.084). The same test remained insignificant in the group with lower late-life SBP (<130 mmHg) (TJT = 37,000; z = 1.639; *p* = 0.101). Similar to SBP, PP ≥ 50 mmHg did not relate to the CAA stage (aOR = 0.89; 95% CI: 0.70–1.14), whereas *APOE* e4 allele carriers (aOR = 1.80; 95% CI: 1.53–2.11) and males (aOR = 1.25; 95% CI: 1.08–1.46) had significantly higher odds of having a more advanced CAA stage than *APOE* e4 non-carriers and female participants, respectively.

The odds of more severe CAA increased with higher Braak and CERAD stages. For example, individuals with “frequent” scores in CERAD (definite AD) were about five times more likely to have more severe CAA (aOR = 5.25; 95% CI: 3.90–7.09) compared to those with no neuritic plaques. Individuals with a Braak stage of V–VI were about 2.43 times more likely to have more severe CAA (95% CI: 1.81 and 3.23) compared to those with a 0–II Braak stage classification. Those with a Braak stage of III–IV have approximately 51% greater odds of more severe CAA (aOR = 1.51; 95% CI: 1.17–1.95) compared to those with a 0–II Braak stage classification.

When the late-life mean PP (Model 1) and late-life mean SBP (Model 2) were excluded from the model, the results were essentially the same.

**Figure 2:**
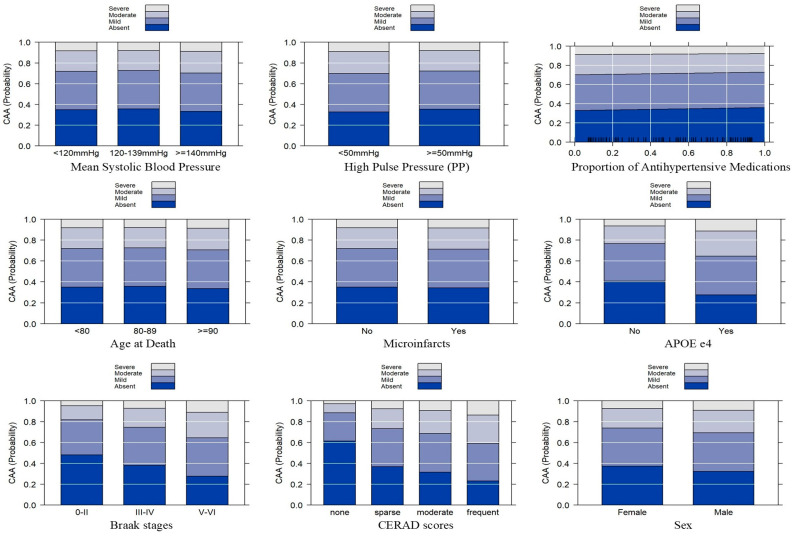
Predictor effect plots with the vertical axis showing a probability scale for the CAA severity stages. The vertical axis “stacks” the probabilities in the four CAA response categories. For example, participants with “no neuritic plaques” (“none” category) for CERAD had nearly 60% probability of having no CAA and about 25% probability of having mild CAA. However, “frequent” neuritic plaques in CERAD scores had approximately 80% probability of having mild to severe CAA with incrementally higher probability with higher CERAD scors. A similar pattern was observed in the Braak predictor effect graph. No differences in CAA severity were apparent for the late-life mean systolic blood pressure predictor effect plot categories or the PP predictor effect plot.

Figure 2 shows predictor effect plots containing the predicted probabilities for each CAA stage across all the variables included in the model. The plot illustrates the effect of the BP categories on CAA severity stages. No differences in the CAA severity were apparent for the late-life mean SBP predictor effect plot categories or the PP predictor effect plot. The trend of increased probability of CAA severity across CERAD and Braak stages is also illustrated in the plots.

## 4. Discussion

Of the participants, 63% had CAA. Moderate and severe CAA cases had a significantly higher proportion of *APOE* e4 carriers. Mild, moderate, and severe CAA cases had incrementally higher severity in the Braak and CERAD stages. Although both the mean SBP and mean PP had no significant association with CAA, the *APOE* e4 status, Braak, CERAD, and males had significant associations with CAA stages. For example, *APOE* e4 carriers had approximately 80% higher odds of greater CAA compared to non-*APOE* e4 carriers. Compared to those with a Braak stage of 0-II, individuals with a Braak stage of III-IV had approximately 51% greater odds of CAA, and those with a Braak stage of V–VI had a 143% higher risk. Similarly, compared to individuals with absent CERAD, those with sparse, moderate, and severe had, respectively, 172%, 246%, and 423% higher risks for CAA. Males had a 26% higher risk for CAA than females. These findings are also apparent in the predictor effect plots (Figure 2).

CAA pathology is very common in older people and increases with age. For example, CAA was present in 38% (320/842) [32] of a general elderly population aged ≥ 65 and 69.6% (213/306) of a general elderly population aged ≥ 85 [33]. Our study is based on a large sample size from 37 past and present ADRCs across the United States. The presence of CAA is not only relevant because of its association with age and with dementia, but also because it can lead to micro-hemorrhages and even macro-hemorrhages, resulting in cognitive impairment and even death [6,34]. For example, in a study of 105 patients with CAA, 54 developed intracranial hemorrhaging that was related to CAA severity: 13% (7/47) grade 1 versus 87% (47/54) grade 2–4 CAA severity [5]. Severe CAA can weaken the vessel wall and cause life-threatening lobar hemorrhages [6]. This becomes even more relevant in the era of anti-amyloid therapy, whose major risks are edema and hemorrhages. Intracranial hemorrhages and cognitive impairment/dementia are closely related [7,8]. In a study of 20 participants with a CAA-related intracranial hemorrhage, 100% of the participants showed mild cognitive impairment after a 4-month follow-up of the intracranial hemorrhage [8].

We found no association between a high late-life mean SBP and mean PP with the CAA stages. Others reported arteriopathy-related small vessel-wall damage from hypertension-related endothelial dysfunction and blood–brain barrier breakdown, leading to the accelerated formation of CAA in rats [13]. Hypertension causes vascular dysfunction (arterial stiffness, increased peripheral resistance, increased arterial pressure, decreased cerebral blood flow, and decreased amyloid β clearance in sequence), leading to increased amyloid β deposition in the parenchyma [35,36]. Data from the Perindopril Protection Against Recurrent Stroke Study reported that lowering the BP resulted in a reduction in intracranial hemorrhages by 46% in people on antithrombotic therapy and by 70% in people without antithrombotic therapy [9]. An increased PP also has a demonstrated association with elevated cortical amyloid burden [13,14] and CSF p-tau levels [37,38]. However, limited empirical studies have examined the association between BP and autopsy-based CAA stages in human beings. To our knowledge, this is the first empirical study looking at the association between BP and CAA. It is possible that the severity and duration of lifetime hypertension have an association with CAA. More studies are needed to validate our findings.

CAA is a condition of amyloid β deposition in the cerebral small vessels measured in this study via autopsy, the gold standard. Since CAA and hypertension are major risk factors for intracranial hemorrhaging [39,40], a high BP may have a synergistic effect on intracranial hemorrhage development in individuals with CAA pathology by making blood vessels with CAA more likely to bleed. However, our study found no association between microbleeds and high BP by CAA severity. More studies are needed to confirm our finding.

Our results demonstrated that *APOE* e4 carriers had higher odds of greater CAA compared to non-*APOE* e4 carriers. The *APOE* e4 allele is a well-known risk factor for Alzheimer’s disease (AD) and CAA stages [41,42,43]. It is believed that *APOE* e4 promotes vascular amyloid accumulation and, thus, increases the odds for moderate or severe CAA [41,42].

Interactions of CAA with neuritic plaque and tau pathology have been found. For example, tau pathology plays a mediating role in the association between CAA and cognitive decline among participants with higher neuritic plaque burden [44]. The UK Biobank data analyses reported that *APOE* e4 carriers had a higher CAA stage and more severe AD pathology (particularly amyloid rather than tau) in cortical regions than non-carriers [45]. A study based on the Rush Religious Orders Study cohort (*n* = 141) reported that people with CAA were more likely to be in Braak stage III than those without CAA (OR, 95% CI: 2.53 (1.06–5.14); *p* = 0.04) [46]. Better understanding of this dynamic interplay may be an essential step for the prevention of CAA formation and progression and, ultimately, the improved management of cognitive impairment and dementia.

Males showed a greater risk for CAA stages than females. Others found similar sex differences in CAA. For example, males with CAA (*n* = 506) had earlier-onset and more hemorrhagic disease than females in the hospital and research databases of the Leiden University Medical Center (2012–2020) and Massachusetts General Hospital (1994–2012) [47]. More confirmatory studies are needed, but researchers assumed possible anti-inflammatory effects of estrogen for the difference [48,49]. Other studies also reported more prevalent and severe CAA cases in males (*n* = 306) [33] and higher overall CAA scores (*n* = 428) [50] than females. In addition, a study based on *n* = 58 patients with CAA found higher medial temporal lobe atrophy in men with dementia compared with women with and without dementia through MRI [51]. Our study offers the largest sample size examining sex-related variations of CAA.

Several limitations of our study need to be acknowledged. Bias due to residual or unmeasured confounders is possible, like in any observational study. We had no data on mid-life BP as well as the severity and duration of lifetime hypertension and could not determine to what extent the observed associations with CAA are attributable to BP in the last decade of life or earlier. The voluntary nature of autopsied participants does not represent all NACC participants. Some Alzheimer’s Disease Research Centers (ADRCs) require that participants agree to autopsy before being accepted for the Uniform Data Set (which contains rich cognitive assessment data that has been captured through annual visits by research experts across the ADRC Program). This may impose further selection pressures on the makeup of the NACC sample. Antihypertensive medication use was based on self-report, and thus, misclassification is possible. The findings from this study, which are based on a predominantly white, over 90% non-Hispanic, urban-dwelling population, may not be generalizable to minority populations. In addition, most ADRCs enroll volunteers with normal cognition, and these tend to be highly educated. Referral-based or volunteer case series of participants may not be representative of the general U.S. population. Lastly, we intended to study AD pathologies (e.g., CERAD, Braak, and *APOE* e4) as covariates in this study and did not separate out dementia with Lewy bodies and frontotemporal dementia. Thus, our findings may be biased to non-AD diagnosis.

## 5. Conclusions

A significant association of the CAA stage was observed with the APOE e4 carrier status, moderate to severe Braak stage (III–IV and V–VI), all stages of CERAD, and the male sex but not with late-life SBP or PP. Future studies need to develop non-invasive laboratory tests to diagnose CAA and prospectively examine the association between BP and CAA and its implication on the pathophysiology and outcome of AD.

## Figures and Tables

**Table 1:** Characteristics of participants by cerebral amyloid angiopathy (CAA) status (*n* = 2510).

Variables	Total Sample (*n* = 2510)		CAA None (*n* = 930)		Mild CAA (*n* = 759)		Moderate CAA (*n* = 529)		Severe CAA (*n* = 292)		*p*-Value ^a^
	N	%	N	%	N	%	N	%	N	%	
**Age at death**											
*<80*	621	24.7%	196	21.1%	184	24.2%	155	29.3%	86	29.5%	**<0.001**
*80–89*	895	35.7%	383	41.2%	266	35.0%	158	29.9%	88	30.1%	
*≥90*	994	39.6%	351	37.7%	309	40.7%	216	40.8%	118	40.4%	
**Apoe e4 carrier**											
*No e4 allele*	1422	56.7%	691	74.3%	409	53.9%	213	40.3%	109	37.3%	**<0.001**
*1 or 2 copies of e4 allele*	1088	43.3%	239	25.7%	350	46.1%	316	59.7%	183	62.7%	
**Braak Stage**											
*0–II*	549	21.9%	372	40.0%	118	15.5%	40	7.6%	19	6.5%	**<0.001**
*III–IV*	724	28.8%	326	35.1%	226	29.8%	126	23.8%	46	15.8%	
*V–VI*	1237	49.3%	232	24.9%	415	54.7%	363	68.6%	227	77.7%	
**CERAD**											
*Absent neuritic plaques*	571	22.7%	417	44.8%	111	14.6%	31	5.9%	12	4.1%	**<0.001**
*Sparse neuritic plaques*	394	15.7%	166	17.8%	132	17.4%	74	14.0%	22	7.5%	
*Moderate*	525	20.9%	165	17.7%	180	23.7%	129	24.4%	51	17.5%	
*Frequent*	1020	40.6%	182	19.6%	336	44.3%	295	55.8%	207	70.9%	
**Race**											
*White*	2363	94.1%	877	94.3%	713	93.9%	496	93.8%	277	94.9%	0.453
*Black or African American*	120	4.8%	44	4.7%	37	4.9%	27	5.1%	12	4.1%	
*American Indian or Alaska Native*	2	0.1%	0	0.0%	1	0.1%	0	0.0%	1	0.3%	
*Asian*	20	0.8%	9	1.0%	7	0.9%	3	0.6%	1	0.3%	
*Other*	5	0.2%	0	0.0%	1	0.1%	3	0.6%	1	0.3%	
**Sex**											
*Female*	1219	48.6%	468	50.3%	385	50.7%	248	46.9%	118	40.4%	**0.012**
*Male*	1291	51.4%	462	49.7%	374	49.3%	281	53.1%	174	59.6%	
**Micro infarcts**											
*No*	1874	74.7%	682	73.3%	567	74.7%	407	76.9%	218	74.7%	0.509
*Yes*	636	25.3%	248	26.7%	192	25.3%	122	23.1%	74	25.3%	
**Mean Late-Life Systolic Blood Pressure**											
*Low: <120 mmHg*	435	17.3%	153	16.5%	135	17.8%	94	17.8%	53	18.2%	0.482
*Normal: 120–139 mmHg*	1335	53.2%	516	55.5%	405	53.4%	272	51.4%	142	48.6%	
*High: ≥140 mmHg*	740	29.5%	261	28.1%	219	28.9%	163	30.8%	97	33.2%	
**Mean Late-Life Pulse Pressure**											
*Normal: <50 mmHg*	487	19.4%	175	18.8%	138	18.2%	103	19.5%	71	24.3%	0.143
*High: ≥50 mmHg*	2023	80.6%	755	81.2%	621	81.8%	426	80.5%	221	75.7%	
	**Mean**	**SD**	**Mean**	**SD**	**Mean**	**SD**	**Mean**	**SD**	**Mean**	**SD**	
**Time gap (last BP to death in years)**	3.029	2.318	2.825	2.296	3.032	2.337	3.217	2.255	3.336	2.395	**0.001**
**Proportion of Antihypertensive Medication Use Across Time**	0.636	0.404	0.679	0.383	0.635	0.404	0.599	0.421	0.567	0.423	**<0.001**

Note: ^a^ *p*-values are from Chi-square tests for categorical variables and ANOVA tests for continuous measures.

**Table 2:** Results of the proportional odds logit model examining the association of late-life mean SBP with cerebral amyloid angiopathy.

Predictor	Model 1 (Excludes Late-Life Mean PP)		Model 2 (Excludes Late-Life Mean SBP)		Model 3 (Includes Both Late-Life Mean SBP and PP)	
	aOR (95% CI)	*p*-Value	aOR (95% CI)	*p*-Value	aOR (95% CI)	*p*-Value
**Late-life mean SBP**						
*120–139 mmHg vs. <120*	0.906 (0.737, 1.115)	0.3510	-	-	0.966 (0.754, 1.238)	0.783
*≥140 mmHg vs. <120*	1.002 (0.797, 1.260)	0.9896	-	-	1.082 (0.816, 1.435)	0.586
**Late-life mean PP**						
*≥50 mmHg vs. <50 mmHg*	-	-	0.910 (0.750, 1.104)	0.3379	0.893 (0.701, 1.138)	0.359
**Proportion of Antihypertensive Medications**	0.869 (0.718, 1.051)	0.056	0.877 (0.725, 1.061)	0.1754	0.872 (0.721, 1.056)	0.160
**Age at Death**						
*80–89 vs. <80*	0.951 (0.783, 1.155)	0.6123	0.966 (0.795, 1.175)	0.7310	0.962 (0.791, 1.170)	0.696
*≥90 vs. <80*	1.055 (0.850, 1.311)	0.6256	1.069 (0.860, 1.329)	0.5480	1.068 (0.859, 1.328)	0.555
**Microinfarcts**						
*Yes vs. No*	1.028 (0.862, 1.224)	0.7612	1.034 (0.868, 1.232)	0.7058	1.028 (0.862, 1.224)	0.762
**APOE e4 carrier**						
*Yes vs. No*	1.795 (1.526, 2.111)	**<0.001**	1.797 (1.528, 2.114)	**<0.001**	1.796 (1.527, 2.113)	**<0.001**
**Braak stage**						
*III–IV vs. 0–II*	1.509 (1.172, 1.943)	**0.0014**	1.513 (1.176, 1.949)	**0.0013**	1.510 (1.173, 1.945)	**0.001**
*V–VI vs. 0–II*	2.427 (1.824, 3.233)	**<0.001**	2.424 (1.821, 3.228)	**<0.001**	2.425 (1.822, 3.230)	**<0.001**
**CERAD stage**						
*Sparse vs. Absent*	2.720 (2.068, 3.583)	**<0.001**	2.744 (2.087, 3.613)	**<0.001**	2.734 (2.079, 3.603)	**<0.001**
*Moderate vs. Absent*	3.456 (2.607, 4.592)	**<0.001**	3.471 (2.619, 4.612)	**<0.001**	3.460 (2.610, 4.598)	**<0.001**
*Frequent vs. Absent*	5.233 (3.885, 7.070)	**<0.001**	5.289 (3.927, 7.145)	**<0.001**	5.251 (3.897, 7.094)	**<0.001**
**Sex**						
*Male vs. Female*	1.255 (1.079, 1.461)	**0.0033**	1.246 (1.071, 1.450)	**0.0044**	1.253 (1.076, 1.458)	**0.004**
Deviance	5881.29		5882.08		5880.45	
AAIC	5913		5912		5914	
Lipsitz goodness- of- fit test (LR statistic, *p*-value)	10.92, *p* = 0.281		8.99, *p* = 0.438		14.487, *p* = 0.110	

## Data Availability

Data can be obtained from https://naccdata.org/ upon request approval.

## Supplementary Materials

The following supporting information can be downloaded at: https://www.mdpi.com/article/10.3390/neurolint16040061/s1, Table S1: Microbleeds and late-life SBP by CAA severity.
