# Supplementary material for: Late-Life Blood Pressure and Cerebral Amyloid Angiopathy: Findings from the U.S. National Alzheimer’s Coordinating Center Uniform Dataset
Source: Neurol Int. 2024 Jul 29;16(4):821–32. doi: 10.3390/neurolint16040061 (PMC11357201; doi:10.3390/neurolint16040061)
Supplement: Supplementary file 1 [file neurolint-16-00061-s001.zip › neurolint-3038632-supplementary.pdf]

Table S1

Microbleeds and late-life SBP by CAA severity

CAA	SBP≥130mmHg				SBP<130mmHg			
	Hemorrhages and Microbleeds				Hemorrhages and Microbleeds			
	Yes	No	Total	Row %	Yes	No	Total	Row %
Absent	35	525	560	6.25	21	406	427	4.92
Mild	25	433	458	5.46	16	303	319	5.02
Moderate	24	289	313	7.67	16	213	229	6.99
Severe	19	158	177	10.73	11	114	125	8.80
